# pH dependencies of glycolytic enzymes of yeast under *in vivo*‐like assay conditions

**DOI:** 10.1111/febs.16459

**Published:** 2022-05-11

**Authors:** Laura Luzia, David Lao‐Martil, Philipp Savakis, Johan van Heerden, Natal van Riel, Bas Teusink

**Affiliations:** ^1^ Systems Biology Lab VU Amsterdam Netherlands; ^2^ Department of Biomedical Engineering TU Eindhoven Netherlands

**Keywords:** enzyme kinetics modeling, nutrient dynamics, pH dependency, progression curve analysis, yeast glycolysis

## Abstract

Under carbon source transitions, the intracellular pH of *Saccharomyces cerevisiae* is subject to change. Dynamics in pH modulate the activity of the glycolytic enzymes, resulting in a change in glycolytic flux and ultimately cell growth. To understand how pH affects the global behavior of glycolysis and ethanol fermentation, we measured the activity of the glycolytic and fermentative enzymes in *S. cerevisiae* under *in vivo*‐like conditions at different pH. We demonstrate that glycolytic enzymes exhibit differential pH dependencies, and optima, in the pH range observed during carbon source transitions. The forward reaction of GAPDH shows the highest decrease in activity, 83%, during a simulated feast/famine regime upon glucose removal (cytosolic pH drop from 7.1 to 6.4). We complement our biochemical characterization of the glycolytic enzymes by fitting the *V_max_* to the progression curves of product formation or decay over time. The fitting analysis shows that the observed changes in enzyme activities require changes in *V_max_*, but changes in *K_m_* cannot be excluded. Our study highlights the relevance of pH as a key player in metabolic regulation and provides a large set of quantitative data that can be explored to improve our understanding of metabolism in dynamic environments.

## Graphical abstract

Under carbon source transitions, *S. cerevisiae* can experience intracellular pH dynamics. Such fluctuations can potentially affect glycolytic fluxes and therefore growth. This work shows how glucose dynamics affect intracellular pH and how glycolytic and ethanol brunch enzyme activity is influenced by pH. Additionally, we developed a modeling approach to extract additional information from the kinetic profiles that allowed us to elaborate on the origin of the different activities.

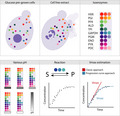

**Abbreviations**
1,3BPG: 1,3‐bisphosphoglyceric acid
2PG: 2‐phosphoglycerate
3PG: 3‐phosphoglycerate
AA: acetaldehyde
ADH: alcohol dehydrogenase
ADP: adenosine diphosphate
ALD: aldolase
ATP: adenosine triphosphate
BCA: bicinchoninic acid assay
BSA: bovine serum albumin
ConA: concanavalin A
DHAP: dihydroxyacetone phosphate
DTT: 1,4‐dithiothreitol
EDTA: ethylenediaminetetraacetic acid
ENO: enolase
F16BP: fructose 1,6‐bisphosphate
F6P: fructose 6‐phosphate
G3PDH: glycerol 3‐phosphate dehydrogenase
G6P: glucose 6‐phosphate, HXK, Hexokinase
GAP: glyceraldehyde 3‐phosphate
GAPDH: glyceraldehyde 3‐phosphate dehydrogenase
GAPDHF: glyceraldehyde 3‐phosphate dehydrogenase forward reaction
GAPDHR: glyceraldehyde 3‐phosphate dehydrogenase reverse reaction
*Keq*
: equilibrium constant
*K_m_*
: Michaelis–Menten constant
MLE: maximum likelihood estimation
NAD: nicotinamide adenine dinucleotide
NADP: nicotinamide adenine dinucleotide phosphate
OD: optical density
ODE: ordinary differential equation
PBS: phosphate‐buffered saline
PDC: pyruvate decarboxylase
PDMS: polydimethylsiloxane
PEP: phosphoenolpyruvate
PFK: phosphofructokinase
PGI: phosphoglucose isomerase
PGK: 3‐phosphoglycerate kinase
PGM: phosphoglycerate mutase
pKa: acid dissociation constant
PYK: pyruvate kinase
PYR: pyruvate
*S. cerevisiae*
: *Saccharomyces cerevisiae*
SSE: sum of squared errors
TPI: triosephosphate isomerase
TTP: thymidine triphosphate
*V_max_*
: maximum reaction rate
YNB: yeast nitrogen base

## Introduction

Microorganisms are constantly challenged by environmental changes, both in nature [1] and in artificial setups such as large‐scale bioreactors [2]. Changes in substrate availability require constant readjustment of metabolism [3, 4] and often rely on allosteric regulation of key metabolic enzymes [5, 6, 7]. Variations in extracellular glucose concentrations have been reported to impact intracellular pH in budding yeast [8, 9, 10]. Other studies linked pH changes with weak acid stress [11, 12], nutrient signaling [13], cell‐cycle progression [14], and growth rate control [15]. In budding yeast, multiple mechanisms are described to contribute to pH homeostasis [16], and a significant amount of cell resources is allocated to this task [17]. Disruption of pH homeostasis can result in changes in enzyme conformation and solubility [18, 19], potentially resulting in the loss of catalytic activity. Additionally, the protonation state of substrates, products, and effectors can be altered [16, 20] and the equilibrium constant of the enzymatic reactions [21].

Several studies seek to describe glycolysis in a systematic fashion [22, 23, 24, 25]; however, kinetic measurements were conducted at a single pH and by design cannot capture the changes in enzyme activities that result in intracellular pH dynamics [26]. Simultaneously, most studies that addressed pH effects of glycolytic enzymes were carried out with purified enzymes and under different experimental conditions, limiting interstudy comparability [11, 27, 28, 29, 30, 31].

**Fig. 1:**
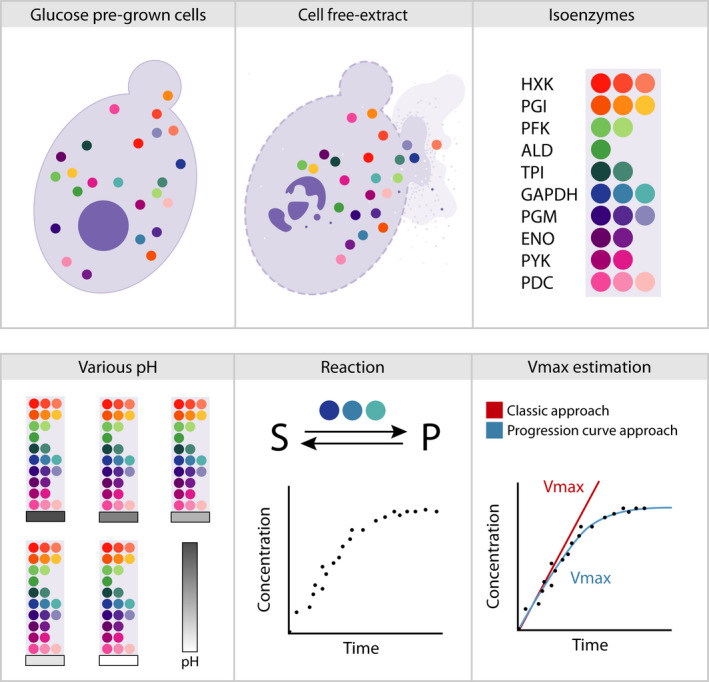
Schematic representation of the experimental design and the computational method developed in this work. Under carbon source transitions, *S. cerevisiae* can experience intracellular pH dynamics. Such fluctuations can potentially affect glycolytic fluxes and therefore growth. This work shows how glucose dynamics affect intracellular pH and how glycolytic and ethanol brunch enzymes activity is influenced by pH. Additionally, we developed a modeling approach to extract additional information from the kinetic profiles that allowed us to elaborate on the origin of the different activities.

In this work, we used the *in vivo*‐like buffer system described by van Eunen *et al.* [23] to measure the enzyme capacities of the glycolytic and fermentative enzymes for the range of pH values often found during nutrient transitions. We focused on glycolysis as this metabolic pathway is an essential part of central carbon metabolism and the main source of ATP and metabolic cell precursors in the presence of fermentative carbon sources. To investigate further the role of the different kinetic parameters in our system, we developed a computational approach that includes the entire progression curve profiles of product formation or decay over time (Fig. 1). By incorporating Michaelis–Menten kinetic parameters such as the Michaelis–Menten constant (*K_m_*) and pH‐dependent equilibrium constant (*K_eq_*) and the variation in metabolite concentrations over time [32, 33, 34], we determined the maximum reaction rate (*V_max_*) [34, 35, 36] using maximum likelihood estimation [37]. Through this method, we obtain parameter estimates, which consider all the available data.

Our study provides a comprehensive description of pH effects on yeast glycolytic and fermentative enzymes. The generated data represents an effort towards more realistic data sets and therefore a step further to understand yeast central carbon metabolism under dynamic glucose environments.

## Results

### Carbon source transitions lead to a rapid cytosolic acidification

**Fig. 2:**
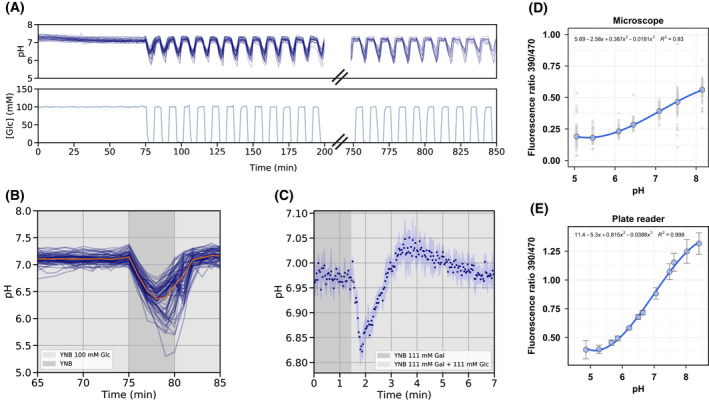
pH dynamics upon glucose transitions. (A) Feast/famine pH dynamics in CEN.PK113‐5D harboring pHluorin performed in a microfluidics device over 850 minutes show a drop in cytosolic pH when glucose is removed. No adaptation was observed over the cycles. Blue lines represent individual cell traces (*n* = 64 at the beginning of the experiment). (B) Zoom in the first cycle of the feast/famine dynamics. The orange line represents the mean of the single‐cell responses. (C) Glucose pulse in galactose pregrown CEN.PK2‐1C cells expressing pHluorin induces a transient drop in pH. Each data point represents the mean of 3 technical replicates (plate reader data). The light blue shaded area represents the standard deviation. pH values reported in (A), (B), and (C) were calculated from fluorescence ratios through *in vivo* calibrations ((D) and (E), for microscope and plate reader titrations, respectively), using polynomial functions of degree 3. Gray dots plotted in (D) represent single‐cell measurements. Error bars in (E) depict the response of three technical replicates.

To estimate the pH changes that follow an increase in glycolytic flux we subjected a population of yeast cells expressing the pH sensor pHluorin [38] to glucose dynamics. We exposed glucose pregrown cells to feast/famine cycles in a microfluidics device by alternating every 5 minutes between media with and without glucose. Using a widefield fluorescence microscope we measured the cytosolic pH dynamics over 850 minutes (about 14 hours) (Fig. 2A). After glucose removal, the cytosolic pH dropped 0.7 pH units (from 7.1 to 6.4, max and min mean pH values, respectively), followed by recovery upon glucose readdition (Fig. 2B). For some cells, pH recovered before glucose readdition, which is likely related to a leakage in the mixing chamber. Nonetheless, all cells within the population showed a similar response, and no adaptation was observed during the cycles. Our results are in line with previous studies that showed that starvation results in cytosolic acidification [15]. Galactose‐adapted cells equally showed a rapid and transient decrease in pH from 6.97 to 6.82 pH units after glucose addition, characterized by an overshoot to slightly above 7, followed by relaxation to preperturbation values (Fig. 2C). To convert the pHluorin ratio to pH, a calibration curve was obtained for both experiments above described using permeabilized cells incubated in a range of pH buffers (Fig. 2D,E).

### Kinetic measurements reveal differential pH sensitivity of glycolytic enzymes

We sought to understand the catalytic behavior of the glycolytic enzymes at different pH values through a series of kinetics measurements under substrate saturation to target *V_max_*. The enzyme activities were measured in cell‐free extracts using coupled enzymatic assays and the reaction rates were monitored through absorbance of NAD(P)H as described by van Eunen *et al.* [23], with the exception of enolase (ENO) reaction that was measured directly by monitoring phosphoenolpyruvate (PEP) absorbance. To calculate the enzyme activities we used the standard method based on the initial slope of the progression curves of substrate production/consumption rate, and a fitting approach here developed that considers the entire progression curves (parameters in Table S1).

**Fig. 3:**
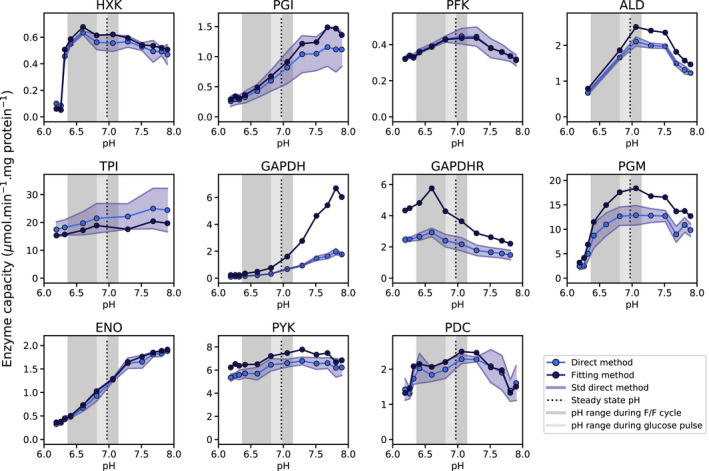
Glycolytic enzymes are pH dependent. *V_max_* estimates obtained for cell‐free extracts of CEN.PK113‐7D with the slope‐based method (dark blue) and the progression curve‐fitting method (light blue) at various pH. The dark gray area corresponds to the pH range of fluctuations observed during the first glucose feast/famine cycle (Fig. 2B) and the light gray area to the pH change observed during the glucose pulse in galactose pregrown cells (Fig. 2C). The dashed line indicates the reference pH 6.8. Each plot describes a glycolytic enzyme: HXK (hexokinase); PGI (phosphoglucose isomerase); PFK (phosphofructokinase); ALD (aldolase); GAPDH (glyceraldehyde 3‐phosphate dehydrogenase forward reaction); GAPDHR (glyceraldehyde 3‐phosphate dehydrogenase reverse reaction); PGM (phosphoglycerate mutase); ENO (enolase); PYK (pyruvate kinase); and PDC (pyruvate decarboxylase). Plots include data of at least 2 dilution factors of the cell‐free extract and 3 technical replicates (Fig. S1 and Table S2 in Supplementary material). The parameters measured by the direct and curve‐fitting approach can be find in Table S1.

**Fig. 4:**
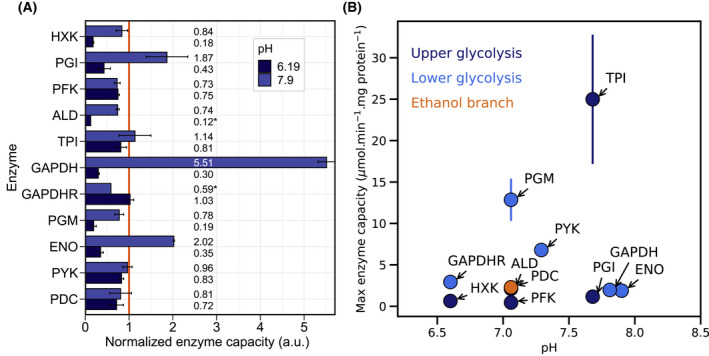
Enzyme activities obtained for the lowest and highest pH under study and maximum enzyme activity of the glycolytic enzymes as a function of pH. (A) Normalized enzyme activities for the lowest and highest pH are represented at dark and light blue, respectively (data normalized to pH 6.8). (*) Represents missing values calculated by fitting the experimental data. (B) Maximum enzyme activity measured for the enzymes under study. The color separates the enzymes by upper glycolysis (dark blue), lower glycolysis (light blue), and ethanol branch (orange). Enzyme activities were estimated using the direct method. (A) and (B) represent data from 3 technical replicates and at least 2 cell‐free extract dilutions (Fig. S1 and Table S2 in Supplementary material).

We found that the rates of all glycolytic enzymes under study were pH‐sensitive, to different degrees (Fig. 3). In the entire pH range surveyed, aldolase (ALD), hexokinase (HXK), phosphoglycerate mutase (PGM), and glyceraldehyde 3‐phosphate dehydrogenase (GAPDH) forward showed the largest decrease in activity from pH 6.8 to 6.2, namely 88, 82, 81 and 70%, respectively (Fig. 4A). By contrast, the rate of the reverse reaction of GAPDH showed a transient increase between pH 6.8 and 6.6, followed by a drop in activity at pH 6.2 to similar values observed at the physiological pH. A more diverse response was observed for higher pH. While the activity of phosphofructokinase (PFK), ALD, GAPDH reverse, and pyruvate decarboxylase (PDC) decreased at higher pH when compared to the reference, it increased for ENO, GAPDH forward, phosphoglucose isomerase (PGI) and triosephosphate isomerase (TPI). The highest change in activity was observed for GAPDH forward, a 6‐fold increase in activity from pH 6.8 to 7.8. Pyruvate kinase (PYK) was the enzyme less affected by pH, with a 16 and 4% decrease in activity from the reference pH to pH 6.2 and 7.9, respectively. No straightforward correlation was observed between pH values at the maximum or optimal enzyme capacity and the enzyme position in the glycolytic pathway (Fig. 4B).

**Fig. 5:**
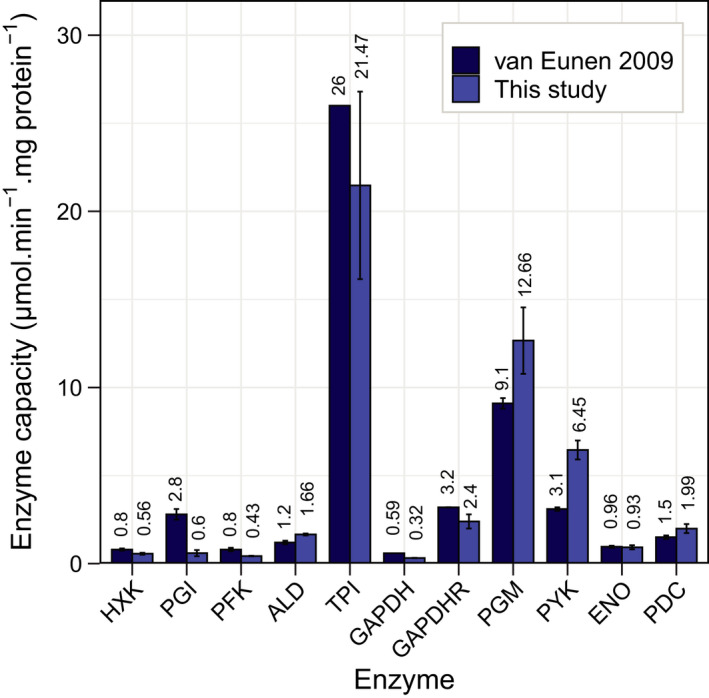
Comparison of enzyme activities at physiological pH from this and an earlier study. Enzyme activities were measured in cell‐free extracts of CEN.PK113‐7D at pH 6.8 using the *in vivo*‐like buffer developed by Eunen [23] (light blue) and compared with the ones obtained from the earlier study (dark blue) under the same conditions (with few exceptions as mentioned in the section Materials and Methods, subsection *in vivo* reactions). Error bars represent the variation of 3 technical replicates and at least 2 cell‐free extract dilutions (This study) and the standard errors of the mean of at least 3 independent cell‐free extracts from samples of a single steady‐state chemostat culture (van Eunen [23]).

We compared the *V_max_* values measured at pH 6.8 (reference pH) to those obtained by van Eunen *et al.* [23] at the same pH (Fig. 5). We found reduced activities (above 70% difference) of PGI and PFK and the forward reaction of GAPDH and an increased activity (above 70% difference) of PYK. These enzymes are among the ones with a larger difference in *V_max_* between reactions performed with different amounts of cell‐free extract (Fig. S1 and Table S2 in Supplementary Material), suggesting the presence of enzyme inhibitors or activators in the reaction mix that could explain the observed discrepancies between the present and the earlier study [23]. Additionally, in the study of van Eunen *et al.* [23], the *V_max_* of PGI and GAPDH in the forward direction were recalculated using measurements in the reverse direction [23] and *K_m_* and *K_eq_* from literature. An important difference between both studies is still related to the growth conditions, while we used exponentially growing cells from a batch culture, van Eunen used chemostat cultures (growth rate of 0.1 *h* ^−1^).

### Enzyme activity estimates based on progression curve‐fitting are in line with those obtained using direct slope estimates

Traditional experimental *V_max_* determination relies on finding the maximal slope of the progression curves of substrate or product concentration over time. However, this method neglects additional information contained in the entire progression curves, such as potential pH effects on *K_m_* and *K_eq_*. Moreover, estimates of slopes are usually performed one at a time, even if the dilution series of the cell‐free extract provide a consistent set of data from which the parameters can be directly obtained. Therefore we determined the maximum reaction rates by generating individual Ordinary differential equation (ODE)‐based models for each reaction. We used single ODEs to model direct enzymatic assays (GAPDH, ENO) and concatenated ODEs for coupled assays. The Teusink model[22] was here used for the glycolytic enzymes under the study following the Michaelis–Menten kinetics [33].

**Fig. 6:**
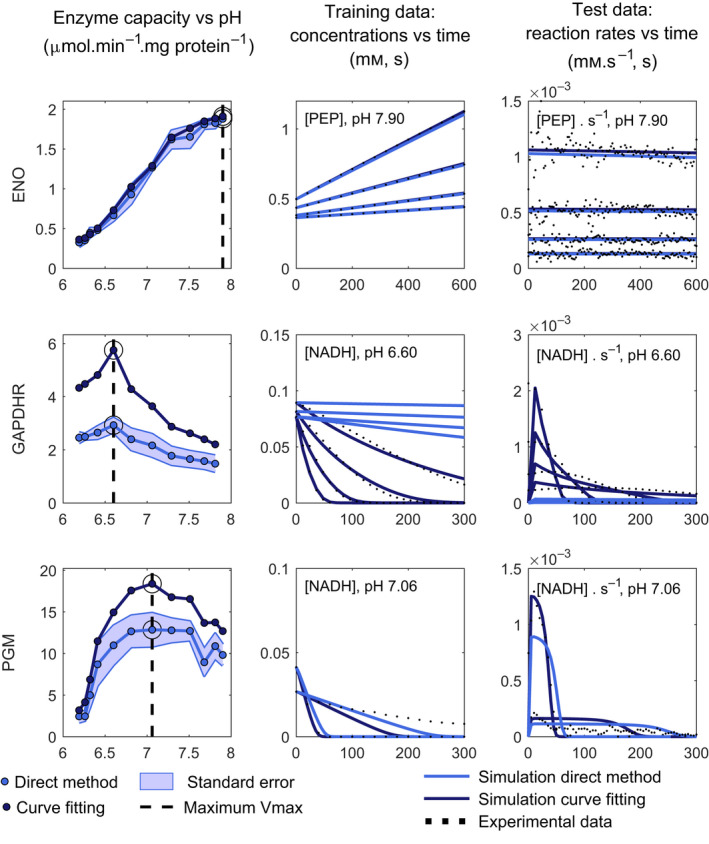
Training data fits can be validated with experimental reaction rates. Examples showing a high performance for ENO and a low performance for GAPDH reverse direction and PGM. Estimates and simulations with the direct and the progression curve‐fitting method are displayed in light and dark blue, respectively. From left to right columns: enzyme capacity estimates, metabolite concentrations, and reaction rate progression curves (training and test data, respectively) at the pH, the maximum activity was measured. The semicontinuous line points represent the pH value at the maximum activity measured and the dots, the experimental data points.

We then proceeded to fit the models' parameters to the kinetic data set generated in this study. Curve‐fitting and slope‐based parameter estimates were in line with the *V_max_* dependency over pH (Fig. 3), i.e., for most enzymes assayed, numerical deviations were within the experimental confidence interval or within 25% of the experimental value at most. The exceptions were PGM and GAPDH, where for GAPDH forward and reverse reactions several computational estimates increased at least by a factor of 2 relative to the slope‐based direct estimates (Fig. 3). The advantage of using both *V_max_* and *K_m_* with our curve‐fitting method, instead of the slope‐based method, becomes clear when simulating the progression curves (Fig. 6). Only when the information contained in the entire progression curves was considered for the estimation of *V_max_*, and *K_m_* was not constrained to the literature values, could the data be accurately described for GAPDH and PGM (Fig. 6, dark blue simulations).

### pH fluctuations during carbon source transitions have a minor influence on driving force

**Fig. 7:**
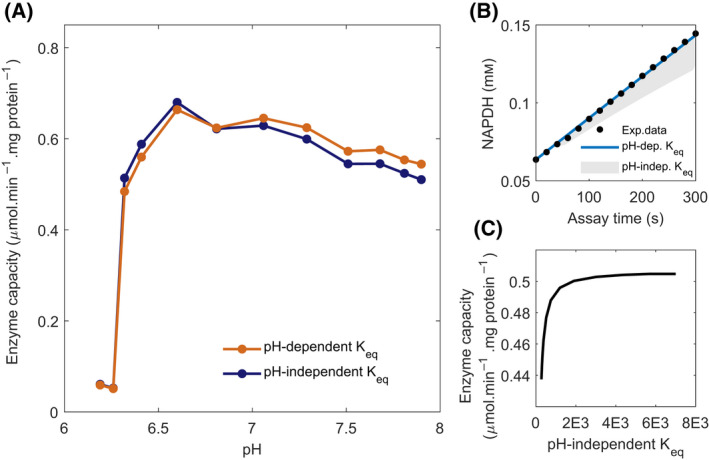
*K_eq_* pH dependence has a limited effect on the *V_max_* estimated for HXK. (A) Effect of pH dependent (dark blue) or pH independent (here fixed at pH 6.8, in orange) *K_eq_* effect on estimated *V_max_*. (B) Simulation of the progression curve at the highest pH value measured (7.90), in comparison with the effect of fixing to the range of *K_eq_* observed in this study (gray‐shaded area, as low as pH 6.19). Dots show the experimental data points. (C) Effect of the different pH‐independent *K_eq_* values (from within the pH range 6.19–7.90) on the *V_max_*.

In reactions that generate or consume protons, pH affects the ratio of the products over substrates at which thermodynamic equilibrium is reached and therefore the equilibrium constant. We estimated the equilibrium constants for the reactions here studied for pH values between 6 and 8, using the eQuilibrator database [39]. The *K_eq_* from enzymes HXK, PFK, GAPDH, PYK, and PDC showed a strong pH dependence for this pH range (2.8‐7.9 × 10^2^ fold change). However, this effect was relatively small in the range of pH values observed across our carbon transitions (2.4‐14 fold change). Next, we investigated whether the effect of pH on some of the *K_eq_* could influence the estimation of the *V_max_* in our assays. We therefore tested both pH‐dependent and pH‐independent *K_eq_* in our models and estimated the *V_max_* for either scenario. With the exception of HXK, the difference was barely noticeable, with *V_max_* estimates differing by a maximum of 10% at higher pH values (Fig. 7). The marginal effect of *K_eq_* on the *V_max_* estimates results from the experimental design of the reactions performed at saturating concentrations of substrates and in the absence of products at the starting of the assay. In this way reactions were kept far from equilibrium, minimizing the effect of *K_eq_* on the reaction rates. Altogether the results confirm the proper design of the enzyme kinetic assays for maximal activity measurements, even though they highlight that pH dependency on *K_eq_* should be included in future models for higher accuracy.

### ODE models reveal pH dependence of *V_max_* for the enzyme enolase

**Fig. 8:**
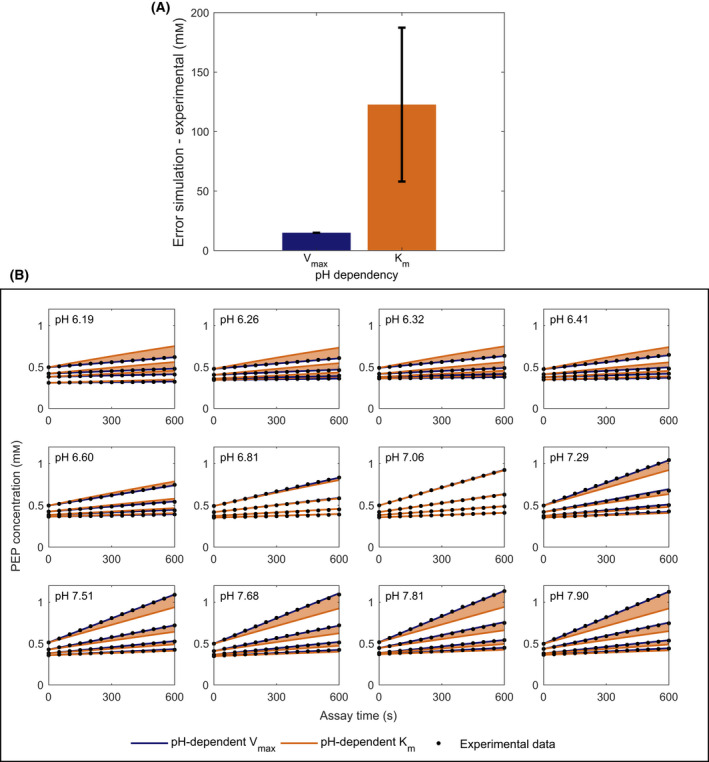
Assuming pH dependence of *V_max_* minimizes simulation error for enolase. (A) Error between simulations and experimental data, consisting of the sum of the concentration profile simulations for all the pH values. (B) Scatter plots showing the progression curves for all the measured pH values at different dilutions of the cell‐free extract. Experimental data points are shown in black, fits with pH‐dependent *V_max_* or *K_m_* in dark blue and orange, respectively. The colored background highlights the increase in error if *V_max_* is fixed to a pH‐independent value.

Michaelis–Menten reactions also depend on *K_m_*, and *K_m_* can depend on pH too. Although the experimental setup was optimized for *V_max_* rather than *K_m_* determination, we tested whether the changes in enzyme activity could be explained by such changes in *K_m_*. To assess this possibility, we used enolase as a case study because the activity of this enzyme can be directly measured by PEP production, without relying on coupling enzymes and therefore reducing uncertainty in the computational analysis. Our analysis confirmed that the parameter *V_max_* changes with pH for the enzyme enolase. The error between simulations and experimental data is smaller when *K_m_* is estimated as a pH‐independent parameter and only *V_max_* is allowed to change with pH (Fig. 8A, blue bar), when compared to the condition where *K_m_* is estimated as a pH‐dependent parameter and *V_max_* is fixed (Fig. 8A, orange bar). The same result can be seen in the progression curve fits (Fig. 8B).

### Carbon source transitions promote a rapid decrease in enzyme activity

**Fig. 9:**
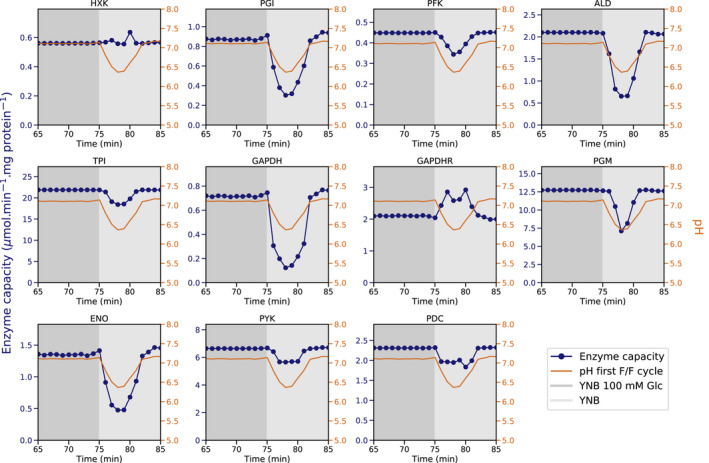
Simulated changes in enzyme activity during a feast/famine cycle. Enzyme activities (dark blue lines) were calculated by interpolating the enzyme activities to changes in pH (orange lines) measured during the first feast/famine cycle (Fig. 2B). Plotted values correspond to the mean values of the individual cell traces.

In this work, we showed that glucose‐adapted cells undergo transient cytosolic acidification when glucose is removed (Fig. 2B) and that changes in cytosolic pH can potentially affect enzyme activities (Fig. 3). To assess the possible effects on enzymes kinetics during glucose dynamics, we extrapolated the changes in enzyme activity from the pH dynamics of the first feast/famine cycle (Fig. 9). Our results show that a decrease in 0.7 pH units (from pH 7.1 to 6.4) can potentially lead to a decrease of 83% activity for the GAPDH forward reaction. In parallel, we observed an increase in activity of 40% for the GAPDH reverse reaction during the removal of glucose. For most enzymes under the study, glycolytic fluxes are compromised during such artificial perturbation with four out of ten enzymes exhibiting a decrease in activity above 60% (PGI, ALD, GAPDH, and ENO).

## Discussion

### Glycolytic enzymes are strongly pH dependent: consequences for the cell

pH is tightly controlled in yeast and simultaneously a highly dynamic property of cell physiology [16, 40]. In this study, we used a glucose feast/famine setup as an example of a dynamic environment that leads to fluctuations in cytosolic pH. The drop in extracellular glucose upon glucose removal leads to an acidification of the cytosol (Fig. 2A), potentially leading to changes in enzyme activities. Variations in intracellular pH in budding yeast have been reported in several studies [8, 15, 17, 40, 41, 42] as a result of both natural and artificial nutrient dynamics. Here we show that the catalytic activity of the glycolytic enzymes is strongly pH dependent, with different enzymes exhibiting distinct degrees of sensitivity (Fig. 3) and optimal pH (Fig. 4B). Simultaneously, no clear cutoff in pH response by upper and lower glycolysis emerged from our study (Fig. 4B). Nonetheless, the large decrease in the activity of GAPDH forward reaction upon glucose depletion (83%) from pH 7.1 to 6.4 could contribute to the previously described imbalance in glycolysis, characterized by a transient accumulation of fructose 1,6‐bisphosphate (F16BP) as upper glycolysis runs faster than the lower glycolysis. Depending on the genetic background, this phenomenon can result in growth arrest [42] and ultimately cell death [31, 43]. The present data identify the GAPDH forward reaction as a potentially problematic step in dynamic pH regimes.

### Cellular mechanisms involved in pH response

There are three potential effects that cytosolic pH changes may have on enzyme activity: (a) changes in enzyme conformation and stability; (b) changes in the ratio of protonated/unprotonated forms of the reactants; and (c) changes in enzyme concentration; or a combination of those [44, 45, 46]. While the first two mechanisms take place in a short time scale (seconds to minutes), the last happens in a longer time scale [7]. Besides ADP (second pKa of 4.99), all compounds used in the assays have a pKa far from the range of pH we tested (https://chemaxon.com), indicating that protonation and deprotonation will not be favored in our experiments and the concentration of reactants is fixed and pH independent. Additionally, possible effects of product inhibition were accounted for by considering reversibility and product concentrations in the modeled systems. Altogether, this suggests that the observed changes in enzyme activity must be enzyme dependent. Those could be due to changes in enzyme conformation, protein aggregation, or changes in the protonation state of the active site, among others.

### The curve‐fitting method adds information contained in the *K_m_* or *K_eq_*

The *V_max_* estimates obtained by the curve‐fitting and the slope‐based direct method are, with few deviations such as for GAPDH and PGM, in agreement (Fig. 3). Here, a better fit was achieved by considering the information contained in the entire progression curves [36], including potential effects of pH on *K_m_* and *K_eq_*, while *K_m_* was not constrained to literature parameters. Furthermore, after implementing pH dependency on the *K_eq_*, it was found that the exclusion of the pH dependency on the *K_eq_* of HXK resulted in a deviation of 10% of the enzyme activities (Fig. 7). Altogether, this suggests that the curve‐fitting method positively complements the slope‐based approach, which might overlook the information contained in *K_m_* or *K_eq_*.

### Including pH dependence in a full glycolysis model could help to explain the response to glucose dynamics

This work shows how intracellular pH changes in response to the addition or removal of glucose and how enzyme activities vary as a function of pH. Enzyme sensitivities were found to vary widely, and GAPDH, previously identified as a flux bottleneck in glycolysis [42], showed the highest pH sensitivity in our assays (Fig. 9). Therefore, our data suggest that future models of glycolysis may need to consider the pH dependencies of enzymes when studying dynamic responses to sudden changes in glucose availability. So far, none of the dynamic models available that studied the cell response to a glucose pulse in yeast have included the pH effect [22, 24, 25, 42].

Since glycolytic enzymes are highly conserved among Eukaryotes that follow the Embden‐Meyerhof‐Parnas pathway [47], it is likely that the activity of these enzymes is also constrained by pH across different species [48]. Interestingly, pH dependency of enzymatic constants *K_m_* and *V_max_* has been successfully implemented in a complete skeletal muscle cell glycogenolysis model, showing the pH dependencies to be necessary to reproduce the physiological kinetics and equilibrium states [49, 50].

## Materials and methods

### 1. Glucose pulse experiment

#### *In situ* pHluorin calibration

pHluorin calibration was performed as described by Smits *et al.* [8] with some modifications. CEN.PK2‐1C (MATa; his3Δ1; leu2‐3112; ura3‐52; trp1‐289; MAL2‐8c; SUC2) was the strain used in the titration. Cells from a single colony were cultivated in bath conditions at 30 °C and 200 rmp in 50 mL of minimal medium containing 6.8 g·L^−1^ Yeast Nitrogen Base (Sigma‐Aldrich, Stl. Louis, MO, USA), 20 mg·L^−1^ L‐histidine (Sigma‐Aldrich), 60 mg·L^−1^ L‐leucine (SERVA Electrophoresis GmbH, Heidelberg, Germany), 20 mg·L^−1^ L‐tryptophan (Sigma‐Aldrich), 1% v/v ethanol (VWR International, Radnor, PA, USA) and 1% v/v glycerol (Sigma‐Aldrich). The OD_600nm_ of the culture was adjusted to 1.5 with YNB buffer (containing 6.8 g·L^−1^ Yeast Nitrogen Base) to a final volume of 40 mL. Cells were washed in 10 mL of the same buffer, followed by 1 min centrifugation at 4000 rpm at room temperature. Cells were collected and resuspend in PBS 10 mm containing 100 µg·mL^−1^ of digitonin (150 µL of the solution was used per OD_600nm_ unit), followed by an incubation of 10 min at 30 °C to allow permeabilization. After incubation cells were collected by centrifugation (5 min at 4000 rpm and 4 °C) and resuspended in PBS 10 mm to an OD_600nm_ = 1.5. The cell suspension was split into tubes (3 mL) and centrifuged twice to collect the PBS (5 min, 4000 rpm followed by 1 min, 4000 rpm at 4 °C). Cells were washed in 1 mL of citric acid/Na_2_HPO_4_ in a range of pH 4.86–8.41 (1 min, 4000 rpm at 4 °C) and resuspended in the same pH buffer. For the titration, cells were diluted to an OD_600nm_ = 0.55 in the final pH buffer, and fluorescence was measured in a 96 well black polystyrene clear‐bottom microplate (Greiner Bio‐One International GmbH, Kremsmünster, Austria). 100 µL of the cell suspension was added per well. Fluorescence excitation was provided at 390 and 470 nm (10 nm bandwidth), and emission was measured at 510 nm using a FLUOstar Omega microplate reader (BMG LABTECH GmbH). The wild‐type strain was used to correct for background fluorescence. The ratio R390/470 was calculated and plotted against the corresponding pH.

#### Glucose pulse

CEN.PK2‐1C cells from a single colony were cultivated in bath conditions at 30 °C and 200 rmp in YNB medium containing 6.8 g·L^−1^ Yeast Nitrogen Base, 20 mg·L^−1^ L‐histidine, 60 mg·L^−1^ L‐leucine, 20 mg·L^−1^ L‐tryptophan and 10.2 g·L^−1^ potassium hydrogen phthalate (VWR International) and supplemented with 111 mm galactose (Sigma‐Aldrich). The pH of the media was adjusted to 5 with KOH (Sigma‐Aldrich). Cells were diluted and collected in mid‐log phase for the pulse experiment. The glucose pulse was performed by the addition of 111 mm glucose (final concentration; Boom BV, Meppel, Netherlands) to the galactose pregrown cells. Excitation at 390 and 470 nm (10 nm bandwidth) was used and emission was measured at 510 nm in a FLUOstar Omega microplate reader. The wild‐type strain was used to correct for background fluorescence. The ratio R390/470 was calculated and converted to pH based on the *In situ* pHluorin calibration above described.

### 2. Feast/Famine experiment

#### *In situ* pHluorin calibration

For the calibration of the pH sensor, pHluorin cells of the strain CENPK.113‐5D (MATa, ura3‐52, HIS3, LEU2, TRP1, MAL2‐8c, and SUC2) were grown in YNB media supplemented with 1% v/v ethanol and 1% v/v glycerol. For the permeabilization step cells were treated as mentioned in Section 1. Glucose pulse experiment, subsection *In situ* pHluorin calibration. For the microscopy imaging, cells were transferred to a Attofluor cell chamber (Thermo Fisher Scientific, Waltham, MA, USA) containing a Concanavalin A (ConA) pretreated coverslip (solution made accordingly by Hansen *et al.* [51]) and incubated for 30 minutes at 30 °C. Before imaging, cells were washed twice with 1 mL of the appropriate pH buffer to remove unattached cells and incubated in 1 mL of the same buffer for imaging. Cells were imaged at 30 °C using a Nikon Ti‐eclipse widefield fluorescence microscope (Nikon, Minato, Tokyo, Japan) equipped with an Andor Zyla 5.5 sCMOS Camera (Andor) and a SOLA 6‐LCR‐SB power source (Lumencor, Beaverton, OR, USA). Fluorescence was recorded using 400/40 nm and 480/40 nm excitation filters, and 505 nm long‐pass and 535/50 nm emission filters (Semrock, Lake Forest, IL, USA). Images were obtained with a Plan Apo λ 100x Oil Ph3 objective (numerical aperture 1.45) using an exposure time of 50 ms and 7.6% of light power. 4 × 4 binning was used to acquire images in the fluorescent channels. Cell segmentation was performed with an in‐house macro using Fiji (NIH, Bethesda, MD, USA).

#### Microfluidics device and time‐lapse microscopy

The strain CENPK.113‐5D expressing pHluorin was used in this experiment. Cells from a single colony were grown in YNB medium supplemented with 100 mm glucose and 10.2 g·L^−1^ potassium hydrogen phthalate (pH adjusted to 5 with KOH) at 30 °C and 200 rpm. Cells were diluted and collected in the mid‐log phase to inject into a PDMS chip attached to a ConA precoated coverslip. The PDMS chip and ConA were prepared as described by Hansen *et al.* [51]. Two reservoirs containing YNB media with and without glucose were connected to a mixing chamber, and the switch between media was performed using a flow rate control module (Maesflow, Fluigent). The flow rates were programmed for an initial 2 hours in glucose followed by 5 minute cycles switch between reservoirs. The feeding flow rate was adjusted to 20 µL·min^−1^. Cells were imaged for 14 hours using the microscopy specification described in Section 2. Feast/Famine experiment, subsection *In situ* pHluorin calibration. The brightfield images were acquired without binning and using 10 ms of exposure time.

#### Cell segmentation and image analysis

Cell segmentation was performed using the brightfield images and a custom in‐house pipeline that uses convolutional networks [52]. The segmentation was performed for each time frame, and the results were used to extract the fluorescence intensity from the fluorescent images. Data were plotted in Python programming language.

### 3. Enzyme kinetics experiments

#### Growth conditions

CENPK.113‐5D was the yeast strain used in this work. Cells from a single colony were grown overnight at 200 rmp and 30 °C in a minimal medium containing 6.8 g·L^−1^ Yeast Nitrogen Base, 20 mg·L^−1^ L‐uracil and 100 mm glucose. Cells were later diluted in 200 mL of the same medium and grown overnight in batch cultivation until they reached 9 generations (mid‐log phase).

#### Cell sampling

Cells were harvested by centrifugation (2500 rpm for 10 min at 4 °C), washed twice in 20 mL of 10 mm potassium phosphate buffer containing 2 mm EDTA (AppliChem GmbH, Darmstadt, Germany) at pH 7.5 (4500 rpm for 5 min at 4 °C) and resuspended in 4 mL of the same buffer 50 times more concentrated. The cell suspension was split into 1 mL aliquots with OD_600nm_ = 40, the equivalent to a dry weight of 14 mg. The supernatant was removed and the cell pellets snap‐frozen in liquid nitrogen and kept at −80 °C for further use.

#### Cell‐free extract preparation

For each enzyme assay, a single‐cell pellet was thawed, washed in 1 mL of 100 mm phosphate buffer containing 2 mm MgCl_2_ (Sigma‐Aldrich) at pH 7.5 and resuspended in an equal volume of the same buffer containing 1 mm 1,4‐Dithiothreitol (DTT; Sigma‐Aldrich). The cell suspension was transferred to a screw cap tube containing 750 mg of acid‐washed glass beads (particle size 425–600 µm; Sigma‐Aldrich) and lysed using a FastPrep‐24 5G (MP Biomedicals, Santa Ana, CA, USA). 8 bursts of 6 m·s^−1^ and 10 s duration were applied to the samples. Between bursts, samples were kept on ice for at least 1 min. The lysates were centrifuged for 15 min at 15000 rpm and 4 °C, and the supernatant was collected for further use.

#### Protein determination

We used the BCA assay to measure the total amount of protein in the cell‐free extracts. The assay was performed according to the specifications of the manufacturer (Pierce BCA Protein Assay Kit, Thermo Scientific). At least two dilutions of the cell‐free extract were used to determine the protein concentration in the sample. DTT was added to the BSA standard to a final concentration of 1 mm to correct for the presence of the compound in the cell‐free extracts.

#### Enzyme kinetic assays

In the present work, we used the standardized *in vivo*‐like buffer developed by van Eunen *et al.* [23] which contains 300 mm potassium, 50 mm phosphate, 245 mm glutamate, 20 mm sodium, 2 mm free magnesium, 2.5–10 mm sulfate, and 0.5 mm calcium at pH 6.8 (reference pH). The pH of the buffer was adjusted by mixing a potassium phosphate buffer (0.85–0.95 M potassium, 0.735 M glutamate, and 0.11 M phosphate) with sodium phosphate (0.51–0.99 M sodium and 0.5 M phosphate) at different ratios. By doing so, we achieved a range of pH from 6.19 to 7.9 and consequently, a change in potassium and sodium composition in the *in vivo*‐like buffer by 11% and 48%, from the lowest to the highest pH, respectively. The free magnesium concentration in the assay was adjusted to 2 mm, considering the presence of cofactors that bind to this ion (ATP, ADP, NADP, NAD, and TTP). The *in vivo*‐like assay medium was stored at 4 °C, and the reaction mix was freshly prepared. The total activity of the isoenzymes was measured at saturating concentrations of substrate and relative to the total amount of protein. The assays were performed according to the reactions described by van Eunen *et al.* [23], with the following exceptions: i) the activity of PFK was measured in the absence of the activator fructose 2,6‐bisphosphate, no longer commercially available; 10 mm of fructose 6‐phosphate (F6P) was used as start chemical, as described in a follow‐up study by van Eunen *et al.* [53]; ii) the activity of TPI was measured with 58 mm glyceraldehyde 3‐phosphate (GAP, 10 times higher concentration), to correct for possible substrate limitation in the reaction mix. The enzyme activity was measured in triplicates and four dilutions of the cell‐free extract were tested per assay to ensure linearity. From those, at least the data of two dilutions were combined in the final analysis (Table S2). The progression of the enzyme reactions was followed by NADH decay or NADPH formation at 340 nm, or PEP formation at 240 nm over time at 30 °C in a SpectraMax Plus 384 (Molecular Devices LLC). The activity of the enzyme mix without start chemical was measured for 5 min, and the rate was used to correct the reaction rate after substrate addition.

#### *In vitro* reactions

**Fig. 10:**
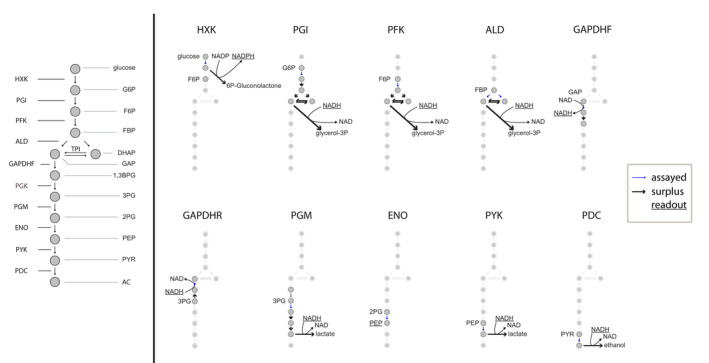
Coupled and direct enzyme assays were employed to measure the kinetics of glycolytic enzymes at different pH values. Thick black arrows denote enzymes that were added in surplus. Annotated molecules indicate that the compound was externally added to the reaction. Underlined molecules denote the metabolites whose concentration changes were measured through absorbance. HXK (hexokinase), PGI (phosphoglucose isomerase), PFK (phosphofructokinase), ALD (aldolase), GAPDHF (glyceraldehyde 3‐phosphate dehydrogenase forward reaction), GAPDHR (glyceraldehyde 3‐phosphate dehydrogenase reverse reaction), PGK (3‐phosphoglycerate kinase), PGM (phosphoglycerate mutase), ENO (enolase), PYK (pyruvate kinase), PDC (pyruvate decarboxylase), F6P (fructose 6‐phosphate), G6P (glucose 6‐phosphate), F16BP (fructose 1,6‐bisphosphate), GAP (glyceraldehyde 3‐phosphate), 3PG (3‐phosphoglycerate), 2PG (2‐phosphoglycerate), PEP (phosphoenolpyruvate), PYR (pyruvate), DHAP (dihydroxyacetone phosphate), 1,3BPG (1,3‐bisphosphoglyceric acid), and AA (acetaldehyde).

*In vitro* reactions were supplemented with purified enzymes and substrates as described below to ensure that every enzyme tested was working at its full capacity. A schematic representation is displayed in Figure 10.

Hexokinase (HXK, EC 2.7.1.1, forward direction): 1 mm NADP, 10 mm glucose, 1 mm ATP, and 1.8 U·mL^−1^ glucose 6‐phosphate dehydrogenase (G6PDH, EC 1.1.1.49).

Phosphoglucose isomerase (PGI, EC 5.3.1.9, forward direction): 0.15 mm NADH, 1 mm ATP, 2.5 U·mL^−1^ phosphofructokinase (PFK, EC 2.7.1.11), 0.45 U·mL^−1^ ALD (EC 4.1.2.13), 0.94 U·mL^−1^ Glycerol 3‐phosphate dehydrogenase (G3PDH, EC 1.1.1.8), 5.5 U·mL^−1^ TPI (EC 5.3.1.1), and 5 mm glucose 6‐phosphate (G6P).

Phosphofructokinase (PFK, EC 2.7.1.11, forward direction): 0.15 mm NADH, 0.5 mm ATP, 10 mm F6P, 0.45 U·mL^−1^ ALD, 0.6 U·mL^−1^ G3PDH, and 1.8 U·mL^−1^ TPI.

Aldolase (ALD, EC 4.1.2.13, forward direction): 0.15 mm NADH, 2 mm F16BP, 0.6 U·mL^−1^ G3PDH, and 1.8 U·mL^−1^ TPI.

Triosephosphate isomerase (TPI, EC 5.3.1.1, forward direction): 0.15 mm NADH, 58 mm GAP, and 8.5 U·mL^−1^ G3PDH.

Glyceraldehyde 3‐phosphate dehydrogenase (GAPDH, EC 1.2.1.12, forward direction): 10 mm ADP, 1 mm NAD, 5.8 mm GAP, and 22.5 U·mL^−1^ 3‐phosphoglycerate kinase (PGK, EC 2.7.2.3).

Glyceraldehyde 3‐phosphate dehydrogenase (GAPDH, EC 1.2.1.12, reverse direction): 1 mm ATP, 0.15 mm NADH, 5 mm 3‐phosphoglycerate (3PG), and 22.5 U·mL^−1^ PGK.

Phosphoglycerate mutase (PGM, EC 5.4.2.1, forward direction): 10 mm ADP, 0.15 mm NADH, 1.25 mm 2,3‐diphospho‐D‐glyceric acid, 5 mm 3PG, 2 U·mL^−1^ enolase (ENO, EC 4.2.1.11), 13 U·mL^−1^ pyruvate kinase (PYK, EC 2.7.1.40), and 11.3 U·mL^−1^ lactate dehydrogenase (LDH, EC 1.1.1.27).

Enolase (ENO, EC 4.2.1.11, forward direction): 6 mm of 2‐phosphoglycerate (2PG).

Pyruvate kinase (PYK, EC 2.7.1.40, forward direction): 10 mm ADP, 0.15 mm NADH, 1 mm F16BP, 2 mm PEP, and 13.8 U·mL^−1^ LDH.

Pyruvate decarboxylase (PDC, EC 4.1.1.1, forward direction): 0.2 mm TPP, 0.15 mm NADH, 50 mm pyruvate, and 88 U·mL^−1^ alcohol dehydrogenase (ADH, EC 1.1.1.1).

The GAPDH forward and ENO reactions were retrieved from van Eunen *et al.* 2009 [53]. The PGM and PGI forward reactions were retrieved from in‐house protocols, written by van Eunen.

#### Enzyme activity

The enzyme activities were calculated using Eqn (1) where the term *ε_NAD(P)H_* (cm^−1^.mM^−1^) × *L*(cm) was replaced by Eqn (2) generated from a calibration curve. A similar approach was used for the enolase Eqn (3).

(1) $$\begin{array}{l} \text{Enzyme capacity}\left(\mu \text{mol}.{\text{min}}^{‐1}.\text{mg}\;{\text{Protein}}^{‐1}\right)\;=\;\frac{NAD\left(P\right)H\;\text{consumptions}\;\left(s^{‐1}\times 60\times \text{dilution factor}\right)}{\left[\text{Protein}\right]\;(\text{mg}.{\text{ml}}^{‐1}\times \varepsilon_{\mathrm{NADH}}\left({\text{cm}}^{‐1}.{\text{mM}}^{‐1}\right)\times L\;\left(\text{cm}\right)\;} \end{array}$$

(2) $$\left[NAD\left(P\right)H\right]\;(\mu \text{mol)}=\frac{\text{Abs}‐0.0628}{4.8365}$$

(3) $$\left[PEP\right]\;\left(\mu \text{mol}\right)=\frac{\text{Abs}+0.008}{1.223}$$

#### Experimental **determination of *V_max_***

The maximum enzyme activity or *V_max_* was experimentally determined from the progression curves (initial measurements while the concentration profiles changed linearly) [32]. The background rate, in the absence of the start chemical, was subtracted from the reaction rates. A conversion factor was applied to correct for sample dilution (60 times from the cell extract to the enzyme assay), and *V_max_* was then expressed as *µ*mol min^−1^ mg Protein^−1^. For all the slope calculations, the size of the data set considered was maximized as long as the R2‐score of the regression remained above 0.995. For some enzymes, estimated values at different dilution factors were not consistent between dilutions. This was the case for TPI and PGI, but also PDC and ALD to a lesser extent. To select which dilution factors were to be considered (Table S2), we implemented a semi‐automated protocol. Briefly, *V_max_* were plotted against the correspondent dilution factor and the data points outside the linear regime were discarded.

#### Kinetic models

Most glycolytic enzymes follow Michaelis–Menten kinetics [32]. Assuming a constant concentration of enzyme, excess of substrate, and absence of products, reversible Michaelis–Menten kinetics can be described by the kinetic parameters: *V_max_*, *K_m_*, and *K_eq_* [33]. The kinetic models used in this work were based on ODEs. Each model, referring to a specific reaction in glycolysis, was expressed by a kinetic expression taken from van Heerden 2009 *et al.* [42]. Most assays consisted of a cascade of reactions that include the coupling reactions and the reaction under study (expressed as concatenated ODEs), with the exception of the directly measured reactions for ENO and GAPDH reverse direction. Parameter values were retrieved from different sources. *V_max_* for the enzymes here investigated were determined in this work. For the coupling reactions, and since all enzymes were in excess, *V_max_* from the literature [22] were increased three orders of magnitude to avoid computational limitations. *K_m_* were obtained from Smallbone *et al.* 2013 [25] and, when not possible, supplemented with data from Teusink *et al.* 2000 [22] and van Heerden 2009 *et al.* [42]. Equilibrium constants were retrieved from the eQuilibrator database[21]. Reaction simulations were performed in Matlab R2017b, and ODEs were manipulated using the tool solver ode15s.

#### Computational estimation of *V_max_*

The analysis of the progression curves was here implemented to estimate the parameters *V_max_* and *K_m_* computationally [34, 36] and the experimental data was used to reparameterize the ODE models generated. For each reaction, a maximum likelihood estimation (MLE) problem was generated considering the optimal cost function as the difference between simulated and experimental data [54]. The sum of squared errors (SSE) problem was solved with the lsqnonlin algorithm in Matlab 2017b. The initial parameter values included the experimentally determined values *V_max_* and *K_m_* from the literature. With the available data, only *V_max_* value could be optimized for each pH value. *K_m_* values were optimized as unique values for the entire pH space. We observed dependency between the *V_max_* and *K_m_* estimates for the enzyme PGM. Therefore, we implemented a regularization factor to penalize deviations in *K_m_* estimates from the literature values [55]. This approach was added to the cost function to reduce the solution space to a realistic region [37].

#### Estimating parameters as constant or changing with pH

To investigate whether *V_max_* and *K_m_* have an influence on the data fitting, we estimated both parameters as pH dependent and independent for the enzyme enolase. When estimating one of the parameters as pH independent and the data could not be fit, the parameter was considered as pH dependent. Equilibrium constants have not been estimated in this work. To assess their influence on *V_max_* estimations, *K_eq_* was fixed to the respective values at the reference pH 6.8 [55].

#### Software used for data analysis, model development, and visualization

Python version 3.8.5 and R Core Team (2013) were used for data analysis and visualization. R: A language and environment for statistical computing. R Foundation for Statistical Computing, Vienna, Austria. ISBN 3‐900051‐07‐0, URL http://www.R‐project.org/. Matlab R2017b was used for model development.

## Conflict of interest

The authors declare no conflict of interest.

### Peer review

The peer review history for this article is available at https://publons.com/publon/10.1111/febs.16459.

## Author contributions

LL, JvH, and BT designed the experiments. LL conducted the kinetics and plate reader experiments. LL and PS conducted the microfluidic experiments. LL, DL, and PS analyzed the results. DL and NvR designed the parameter estimation analysis. DL performed the parameter estimation analysis. LL, DL, and PS wrote the manuscript. BT and NvR supervised the research. All authors reviewed the manuscript.

## Supporting information


**Fig. S1**. Enzyme capacities for the enzymes were tested at different dilution factors. Color intensity indicates the dilution factor (DF) of the cell‐free extract. Four dilution factors were tested for each enzyme.
**Table S1**. Parameters are measured by the direct and curve‐fitting approach. Enzyme capacities ( mol min^−1^. mg Protein^−1^) are displayed for each pH value assayed. Curve‐fitting estimates (simulation, sim), direct determinations (experimental, exp), difference between curve‐fitting estimates, and direct determinations, in percentage (diff).
**Table S2**. Dilution factors were selected for *V_max_* estimation for each enzyme and pH. Dilution factors range from 1 to 32.

## Data Availability

The raw data, scripts, and models are available in the github repository https://github.com/DavidLaoM/pH_kinetics.
