# Supplementary material for: pH dependencies of glycolytic enzymes of yeast under in vivo‐like assay conditions
Source: FEBS J. 2022 May 11;289(19):6021–37. doi: 10.1111/febs.16459 (PMC9790636; doi:10.1111/febs.16459)
Supplement: Supplementary file 1 — Fig. S1. Enzyme capacities for the enzymes were tested at different dilution factors. Color intensity indicates the dilution factor (DF) of the cell‐free extract. Four dilution factors were tested for each enzyme. Table S1. Parameters are measured by the direct and curve‐fitting approach. Enzyme capacities ( mol min−1. mg Protein−1) are displayed for each pH value assayed. Curve‐fitting estimates (simulation, sim), direct determinations (experimental, exp), difference between curve‐fitting estimates, and direct determinations, in percentage (diff). Table S2. Dilution factors were selected for Vmax estimation for each enzyme and pH. Dilution factors range from 1 to 32. [file FEBS-289-6021-s001.zip › febs16459-sup-0001-Supinfo.pdf]

FEBS PRESS
science publishing by scientists
WILEY
The FEBS Journal
www.febsjournal.org

pH dependencies of glycolytic enzymes of yeast under *in vivo*-like assay conditions

Laura Luzia, David Lao-Martil, Philipp Savakis, Johan van Heerden, Natal van Riel and Bas Teusink

DOI: 10.1111/febs.16459

Contents

1 Comparison of experimental and computationally determined V_{max} 2
2 Dilution factors used in enzymatic assays 4

1 Comparison of experimental and computationally determined V_{max}

Table S1. Parameters measured by the direct and curve fitting approach. Enzyme capacities (µmol. min^{-1}. mg $Protein^{-1}$) are displayed for each pH value assayed. Curve fitting estimates (simulation, sim), direct determinations (experimental, exp), difference between curve fitting estimates and direct determinations, in percentage (diff)

pH	HXK			PGI			PFK			ALD		
	sim	exp	diff	sim	exp	diff	sim	exp	diff	sim	exp	diff
6.19	0.06	0.10	40.18	0.29	0.26	12.44	0.32	0.32	0.54			
6.26	0.05	0.08	38.98	0.34	0.29	16.41	0.34	0.34	0.20			
6.32	0.51	0.46	11.49	0.31	0.29	8.82	0.33	0.33	1.28	0.79	0.67	17.83
6.41	0.59	0.55	6.57	0.36	0.33	10.54	0.36	0.36	1.80			
6.60	0.68	0.63	6.80	0.49	0.43	13.67	0.39	0.39	1.70			
6.81	0.61	0.56	9.26	0.67	0.60	12.21	0.43	0.43	0.65	1.86	1.66	12.48
7.06	0.62	0.56	11.85	0.91	0.82	11.60	0.44	0.45	2.39	2.51	2.11	19.31
7.29	0.59	0.57	4.86	1.21	1.04	16.72	0.44	0.44	1.76	2.41	1.99	21.06
7.51	0.54	0.53	2.43	1.24	1.05	18.34	0.38	0.38	0.43	2.36	1.97	19.53
7.68	0.54	0.49	8.47	1.49	1.16	28.41	0.36	0.36	0.23	1.82	1.49	21.71
7.81	0.52	0.49	5.76	1.47	1.12	31.19	0.34	0.34	0.11	1.57	1.31	20.02
7.90	0.51	0.47	8.07	1.36	1.12	21.45	0.32	0.31	1.59	1.47	1.23	19.76

pH	TPI			GAPDH			GAPDHR			PGM		
	sim	exp	diff	sim	exp	diff	sim	exp	diff	sim	exp	diff
6.19	15.31	17.43	12.17	0.23	0.10	140.23	4.33	2.46	76.32	3.18	2.44	30.22
6.26				0.23	0.12	92.57	4.48	2.49	79.77	4.14	2.45	69.27
6.32	15.68	18.21	13.90	0.23	0.10	116.73				6.87	4.99	37.62
6.41				0.35	0.15	125.27	4.82	2.65	81.66	11.48	8.70	32.04
6.60	17.19	19.69	12.71	0.48	0.22	121.82	5.75	2.93	96.45	14.94	10.98	36.08
6.81	18.89	21.47	12.04	0.76	0.32	136.75	4.28	2.40	78.69	17.57	12.66	38.78
7.06				1.60	0.67	138.93	3.64	2.17	68.13	18.38	12.85	43.01
7.29	17.55	22.14	20.73	2.77	0.93	196.00	2.87	1.78	61.60	16.77	12.79	31.14
7.51				4.63	1.47	215.34	2.62	1.65	58.62	16.55	12.71	30.17
7.68	20.41	24.98	18.31	5.42	1.63	233.14	2.40	1.58	52.21	13.67	8.94	52.84
7.81				6.69	1.97	239.33	2.21	1.48	49.16	13.74	10.89	26.15
7.90	19.69	24.40	19.32	6.03	1.77	241.40				12.69	9.82	29.22

pH	ENO			PYK			PDC		
	sim	exp	diff	sim	exp	diff	sim	exp	diff
6.19	0.36	0.33	11.63	6.23	5.35	16.33	1.32	1.43	7.22
6.26	0.38	0.35	7.85	6.53	5.50	18.60	1.46	1.31	11.77
6.32	0.43	0.45	4.34	6.37	5.58	14.17	2.08	1.73	20.31
6.41	0.50	0.48	4.59	6.47	5.71	13.30	2.15	2.03	6.09
6.60	0.73	0.67	9.50	6.51	5.69	14.34	2.07	1.84	12.24
6.81	1.03	0.93	10.45	7.21	6.45	11.78	2.21	1.99	10.77
7.06	1.29	1.27	1.71	7.47	6.60	13.14	2.50	2.29	9.38
7.29	1.65	1.62	1.59	7.77	6.79	14.47	2.47	2.28	8.46
7.51	1.76	1.65	6.52	7.34	6.56	11.79	2.05	2.09	1.73
7.68	1.85	1.81	2.16	7.47	6.60	13.17	1.97	1.90	3.68
7.81	1.89	1.83	3.16	6.69	6.19	8.01	1.33	1.39	4.59
7.90	1.91	1.88	1.70	6.85	6.22	10.05	1.50	1.60	6.26

2 Dilution factors used in enzymatic assays

Some reactions showed different rates for the cell-free extract dilution tested, with some enzymes showing a higher activity at higher dilutions (PGI and TPI) while others showed a higher activity at lower dilutions (PFK and PDC). The dilution factors used to calculate enzyme capacities differed sometimes per enzyme and pH tested. Figure 1 displays the enzyme capacities calculated with the direct method. For case of inconsistency between cell-free extracts dilutions, the least diluted samples were usually considered. The reaction rates experimentally calculated for each enzyme and pH are displayed in figure 1. Table 2 shows the dilution factors values considered.

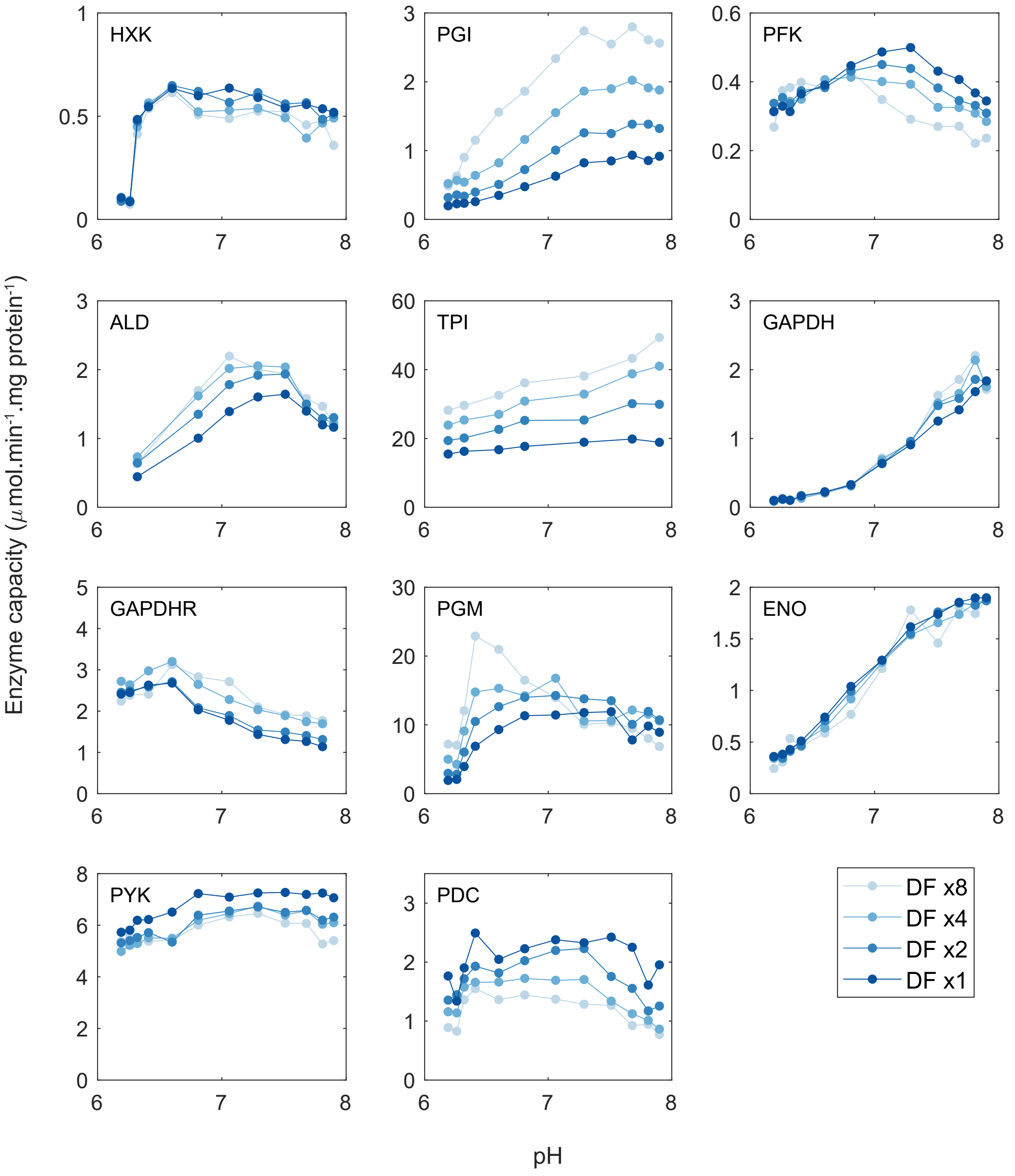

Figure S1. Enzyme capacities for the enzymes tested at different dilution factors. Color intensity indicates the dilution factor (DF) of the cell free-extract. Four dilution factors were tested for each enzymes.

Table S2. Dilution factors selected for V_{max} estimation for each enzyme and pH. Dilution factors range from 1 to 32.

pH	HXK	PGI	PFK	ALD	TPI	GAPDH	GAPDHR	PGM	ENO	PYK	PDC
6.19	8 4 2 1	- - 2 1	16 8 4 -	- - - -	16 8 - -	16 8 4 2	8 4 2 1	- - 4 2	8 4 2 1	16 8 4 2	- 4 2 1
6.26	8 4 2 1	- - 2 1	16 8 4 -	- - - -	- - - -	16 8 4 2	8 4 2 1	- - 4 2	8 4 2 1	16 8 4 2	- 4 2 1
6.32	8 4 2 1	- - 2 1	16 8 4 -	8 4 2 -	16 8 - -	16 8 4 -	- - - -	- 8 4 -	8 4 2 1	32 16 8 4	- 4 2 1
6.41	8 4 2 1	- - 2 1	16 8 4 -	- - - -	- - - -	8 4 2 1	8 4 2 1	16 8 - -	8 4 2 1	32 16 8 4	- 4 2 1
6.60	8 4 2 1	- - 2 1	16 8 4 -	- - - -	16 8 - -	8 4 2 1	8 4 2 1	16 8 - -	8 4 2 1	32 16 8 4	- 4 2 1
6.81	8 4 2 1	- - 2 1	16 8 4 -	8 4 - -	16 8 - -	8 4 2 1	8 4 2 1	16 8 - -	8 4 2 1	32 16 8 4	- 4 2 1
7.60	8 4 2 1	- - 2 1	16 8 4 -	8 4 - -	- - - -	8 4 2 1	8 4 2 1	16 8 - -	8 4 2 1	32 16 8 4	- - 2 1
7.29	8 4 2 1	- - 2 1	16 8 4 -	8 4 2 -	16 8 - -	8 4 2 1	8 4 2 1	16 8 - -	8 4 2 1	32 16 8 4	- - 2 1
7.51	8 4 2 1	- - 2 1	16 8 4 -	8 4 2 -	- - - -	8 4 2 1	8 4 2 1	16 8 - -	8 4 2 1	32 16 8 4	- - 2 1
7.68	8 4 2 1	- - 2 1	16 8 4 -	8 4 2 1	16 8 - -	8 4 2 1	8 4 2 1	- 8 4 -	8 4 2 1	32 16 8 4	- - 2 1
7.81	8 4 2 1	- - 2 1	16 8 4 -	8 4 2 1	- - - -	8 4 2 1	8 4 2 1	- 8 4 -	8 4 2 1	32 16 8 4	- - 2 1
7.90	8 4 2 1	- - 2 1	16 8 4 -	8 4 2 1	16 8- -	16 8 4 -	- - - -	- 8 4 -	8 4 2 1	32 16 8 4	- - 2 1
